# The Interaction of Apelin and FGFR1 Ameliorated the Kidney Fibrosis through Suppression of TGF*β*-Induced Endothelial-to-Mesenchymal Transition

**DOI:** 10.1155/2023/5012474

**Published:** 2023-02-04

**Authors:** Rongfen Gao, Yumin Wu, Qian Yang, Liangdong Chen, Jiwei Chen, Boyong Wang, Zeming Liu, Juan Jin, Jinpeng Li, Gaosong Wu

**Affiliations:** ^1^Department of Thyroid and Breast Surgery, Zhongnan Hospital of Wuhan University, Wuhan 430000, China; ^2^Department of Rheumatology and Immunology, Tongji Hospital, Tongji Medical College, Huazhong University of Science and Technology, Wuhan 430000, China; ^3^Department of Plastic and Cosmetic Surgery, Tongji Hospital, Tongji Medical College, Huazhong University of Science and Technology, China; ^4^Department of Nephrology, Zhejiang Provincial People's Hospital, Affiliated People's Hospital, Hangzhou Medical College, Hangzhou 310000, China

## Abstract

Both epithelial-to-mesenchymal (EMT) and endothelial-to-mesenchymal (EndMT) transitions have shown to contribute to the development and progression of kidney fibrosis. It has been reported that apelin, a regulatory peptide, alleviates EMT by inhibiting the transforming growth factor *β* (TGF*β*) pathway in renal diseases. Additionally, fibroblast growth factor receptor 1 (FGFR1) has been shown to be a key inhibitor of EndMT through suppression of the TGF*β*/Smad pathway. In this study, we found that apelin and FGFR1 were spatially close to each other and that the apelin and FGFR1 complex displayed inhibitory effects on TGF*β*/Smad signaling as well as associated EndMT in diabetic kidney fibrosis. In cultured human dermal microvascular endothelial cells (HMVECs), we found that the anti-EndMT and anti-TGF*β*/Smad effects of apelin were dampened in FGFR1-deficient cells. Either siRNA- or an inhibitor-mediated deficiency of apelin induced the Smad3 phosphorylation and EndMT. Streptozotocin-induced CD-1 diabetic mice displayed EndMT and associated kidney fibrosis, which were restored by apelin treatment. The medium from apelin-deficient endothelial cells stimulated TGF*β*/Smad-dependent EMT in cultured HK2 cells. In addition, depletion of apelin and the FGFR1 complex impaired CEBPA expression, and TGF*β*-induced repression of CEBPA expression contributed to the initiation of EndMT in the endothelium. Collectively, these findings revealed that the interaction between apelin and FGFR1 displayed renoprotective potential through suppression of the TGF*β*/Smad/CEBPA-mediated EndMT/EMT pathways.

## 1. Introduction

Kidney fibrosis represents the end-stage of all types of progressive chronic kidney disease (CKD). However, there is a lack of suitable strategies to reverse fibrosis in kidneys [1–3]. Previous studies have revealed that several mechanisms, including EMT and EndMT, are involved in the development and progression of renal fibrosis [4–6]. EMT is a biological repair process in which epithelial characteristics are lost, and mesenchymal markers are acquired. EndMT is a subtype of EMT in which endothelial cells lose characteristics such as smooth muscle *α*-actin (*α*-SMA), vimentin, and smooth muscle protein 22 *α* (SM22*α*) and gain mesenchymal characteristics such as VE-cadherin and CD31 (also known as platelet endothelial cell adhesion molecule-1) [7]. EMT and EndMT influence each other in the diabetic kidney and accelerate fibrosis [8, 9]. EndMT is known to be associated with a variety of pathological changes, such as myocardial, pulmonary, and renal fibrosis, cancer proliferation and metastasis, and diabetic nephropathy [9–12]. Endothelial cells are stimulated by various cytokines, including transforming growth factor *β* (TGF*β*), fibroblast growth factor (FGF), and interferon gamma (INF-*γ*) to undergo EndMT [11]. Overall, it has been shown that TGF*β* is the main stimulant which induces EndMT through suppression of Smad3 activation [13–15].

Apelin is a regulatory peptide involved in various biological processes, including cardiovascular and fluid homeostasis, inflammation, cell proliferation, angiogenesis, and hepatic fibrosis [16, 17]. Apelin 13, identified as the major isoform [18], is a potent angiogenic regulator that activates the endothelial apelin receptor (APJ) [19, 20]. The apelin/APJ system is involved in the regulation of diabetes and diabetic complications such as diabetic kidney disease [16, 21–24]. Many medications have recently been found to improve EndMT and kidney fibrosis. For example, DPP-4, which interacts with integrin*β*1, may lead to EndMT [25]. Furthermore, the DPP-4 inhibitor linagliptin inhibits EndMT and fibrosis [26]. Additionally, ACE inhibitors and the bioavailable peptide AcSDKP inhibit EndMT by inhibiting DPP-4 levels [27]. Previous studies have revealed that several mechanisms, including EMT and EndMT, are also involved in the development and progression of organ fibrosis [28–31]. Furthermore, emerging evidence demonstrates that apelin reduces EMT by targeting TGF*β* in renal diseases [32, 33]. However, the molecular mechanisms underlying the effect of apelin on endothelial cells are poorly understood.

FGFR signaling plays a crucial role in various physiological processes, such as tissue and metabolism homeostasis, endocrine functions, and differentiation and EMT of their target cells [34, 35]. A previous study reported that apelin is related to aberrant expression of FGFR1 in the endothelial cells of the pulmonary arteries [36]. It was observed that apelin could attenuate renal fibrosis by suppressing the EMT of podocytes and tubules through TGF*β*/Smad signaling [32, 33]. There is evidence that the inhibition of FGFR1 activates TGF*β* signaling and induces EndMT [37–39]. Thus, it was assumed for the sake of this study that the interaction between apelin and FGFR1 suppresses TGF*β*-induced EndMT in the progression of kidney fibrosis.

## 2. Materials and Methods

### 2.1. Reagents and Antibodies

The apelin peptide (ab152927) was purchased from Abcam (Cambridge, UK). The apelin inhibitor (ML221) (HY-103254) and recombinant human transforming growth factor *β*2 (HY-P7119) were purchased from MedChemExpress LLC (New Jersey, USA). Human neutralizing FGFR1 (MAB765) was obtained from R&D Systems (Minneapolis, MN, USA). Phosphor-Smad3-S423/S425 rabbit monoclonal antibody (AP0727) and vimentin rabbit monoclonal antibody (A19607) were obtained from ABclonal Technology (Wuhan, China). The following were obtained from Wuhan Sanying (Wuhan, China): E-cadherin rabbit polyclonal antibody (20874-1-AP), Smad3 rabbit polyclonal antibody (25494-1-AP), CEBPA polyclonal antibody (18311-1-AP), *β*-actin mouse monoclonal antibody (66009-1-lg), and GADPH rabbit monoclonal antibody (60004-1-lg). Mouse monoclonal anti-FGFR1 (ab824) and rabbit polyclonal anti-TGF*β*R2 (ab61213) antibodies were obtained from Abcam (Cambridge, UK). Rabbit anti-TGF*β*R1 (SAB4502958) antibody was purchased from Sigma-Aldrich (St. Louis, MO, USA). *α*-SMA rabbit monoclonal antibody was purchased from Cell Signaling Technology (Danvers, MA, USA). Rabbit polyclonal anti-SM22*α* antibody was obtained from Novus Biologicals (Littleton, CO, USA). Rabbit polyclonal anti-S100A4 (also known as FSP1) (PRB-497P), mouse monoclonal anti-VE-cadherin (sc-9989), and PECAM-1 (also known as CD31) (sc-365804) were purchased from Santa Cruz Biotechnology (Dallas, TX, USA).

### 2.2. Cell Culture and Treatment

The human dermal microvascular endothelial cells (HMVECs) were cultured in EBM-2 medium supplemented with EGM-2 which contained fetal bovine serum, 5.5 mmol/L-glucose, hydrocortisone, VEGF, R-IGF-1, hFGF-*β*, ascorbic acid, GA-1000, hEGF, and heparin (Lonza, Alpharetta, GA, USA). When the cells reached 70-80% confluence, TGF*β*2 (5 ng/mL), N-FGFR1 (1.5 *μ*g/mL), apelin (100 nM), or ML221 (10 *μ*M) was added to the experimental medium (a mixture of HuMedia-MVG in serum-free RPMI 1640 medium at a 1 : 3 ratio).

Human HK-2 cells were cultured in MEM supplemented with 10% fetal bovine serum (Invitrogen, Carlsbad, CA, USA). To establish the conditioned medium experiment [8], HMVECs were transfected with scramble or apelin siRNA for 6 h, after which the medium was replaced with fresh EBM-2 medium. After incubation with fresh medium for 48 h, the experimental medium of HMVECs was harvested and transferred to cultured HK2 cells.

### 2.3. Transfection Experiments

HMVECs were transfected with siRNA (100 nmol/L) targeting apelin and CEBPA. The apelin siRNA sequences were 5′-GCAUCCCAAAUCGGUUCUATT-3′ and 3′-UAGAACCGAUUUGGGAUGCTT-5′. The CEBPA siRNA sequence was 5′-CCUUCAACGACGAGUUCCUTT-3′ and 3′-AGGAACUCGUCGUGAAGGTT-5′. For transient transfection of siRNAs, cells at ~60% confluence were transfected with Lipofectamine RNAiMAX (13778100, Invitrogen).

### 2.4. Duolink *In Situ* Assay

The manufacturer's protocol for the Duolink *in situ* proximity ligation assay (PLA) was followed. HMVECs were cultured with TGF*β*2 (5 ng/mL) or N-FGFR1 (1.5 *μ*g/mL) for 48 h with and without apelin preincubation. After fixing with 4% paraformaldehyde, cells were permeabilized with 0.2% Triton X-100. After blocking, the cells were treated with rabbit antiapelin and mouse anti-FGFR1 antibodies overnight. The cells were incubated for 1 h at 37°C with the PLA probe solution before being treated with ligase solution for 30 min at 37°C and a polymerase amplification solution for 100 min at 37°C. The samples were promptly mounted for 20 min with Duolink *in situ* mounting medium containing DAPI, and fluorescence microscopy was used for the examination. The images were evaluated from six separate view fields at ×400 magnification for each slide.

### 2.5. Western Blot Analysis

RIPA lysis buffer (ABclonal Technology Co., Wuhan, China) was used to lyse nuclear and cytoplasmic proteins. Protein lysates were boiled for 10 min at 100°C in SDS sample buffer and transferred to PVDF membranes after separation on SDS-polyacrylamide gels. Next, 5% skim milk was used to block the mixture. After blocking, the membranes were incubated overnight at 4°C with primary antibodies, followed by incubation with secondary antibodies for 1 h at room temperature. Blots were analyzed using a chemiluminescence imaging system (Tanon, Shanghai, China).

### 2.6. Immunofluorescence for Cell Culture

The treated HMVECs were plated on six-well culture slides (354630, BioCoat) for 48 h. 100% methanol was used to fix the cells for 10 min at -20°C, which was followed by acetone for 1 min at -20°C. After that, the cells were blocked with 2% bovine serum albumin (BSA) for 30 min at room temperature and then incubated with the primary antibody, followed by binding to the corresponding secondary antibodies for 30 min. Finally, PBS was used to thoroughly wash the mixture three times, and a mounting medium containing DAPI was used to mount it. A fluorescence microscope (Axio Vert. A1, Carl Zeiss Microscopy GmbH) was used to visualize all images.

### 2.7. Immunostaining Analysis of Mouse Tissues

Briefly, acetone was used to fix the frozen kidney sections for 10 min at -20°C. The sections were blocked with 2% BSA for 30 min at room temperature, followed by incubation with primary antibodies against CD31/FSP1, CD31/*α*-SMA, and CD31/vimentin. The corresponding secondary antibodies were used to incubate the sections successively. The mounting medium containing DAPI was then mounted for 10 min at room temperature. A fluorescence microscope (Axio Vert. A1, Carl Zeiss Microscopy GmbH) was used to visualize all images.

### 2.8. Morphological Evaluation

PAS-stained glomeruli from each mouse were detected using a digital microscope screen grid containing 540 (27 × 20) points. Based on Masson's trichrome-stained and Sirius red-stained tissue images of each section, fibrotic areas were evaluated. Six separate fields from each mouse were analyzed at ×40 magnification.

### 2.9. Animal Experiments

Male CD-1 mice (4 weeks old) were obtained from Beijing Vital River Laboratory Animal Technology Co., Ltd. (Beijing, China) and randomly divided into control, STZ-treated CD-1, and apelin-treated STZ mice, according to a previous study [38, 40]. Briefly, 8-week-old CD-1 mice were intraperitoneally injected with streptozotocin (STZ; 200 mg/kg). Sixteen weeks after the induction of diabetes, the STZ-treated mice were assigned to the nontreatment group or the apelin treatment group (500 *μ*g/kg BW/day using an osmotic minipump for 8 weeks). Animal experiments were performed in the laboratory animal facility of Zhongnan Hospital of Wuhan University according to the requirements of animal ethics and were approved by the Laboratory Animal Management and Use Committee of the Animal Experiment Center of Wuhan University (WP2020-08103).

### 2.10. Statistical Analysis

Statistical analyses were conducted using GraphPad Prism software (version 8.0). Data are expressed as mean ± SD of at least three independent experiments. Two group comparisons were performed using an unpaired two-tailed *t-*test (statistical significance was defined as *P* < 0.05). Ns indicates nonsignificant (^∗^*p* < 0.05, ^∗∗^*p* < 0.01, ^∗∗∗^*p* < 0.001, and ^∗∗∗∗^*p* < 0.0001).

## 3. Results

### 3.1. Proximity of Apelin and FGFR1 for the Suppression of TGF*β*/Smad Signaling Pathway in the Endothelium

**Figure 1:**
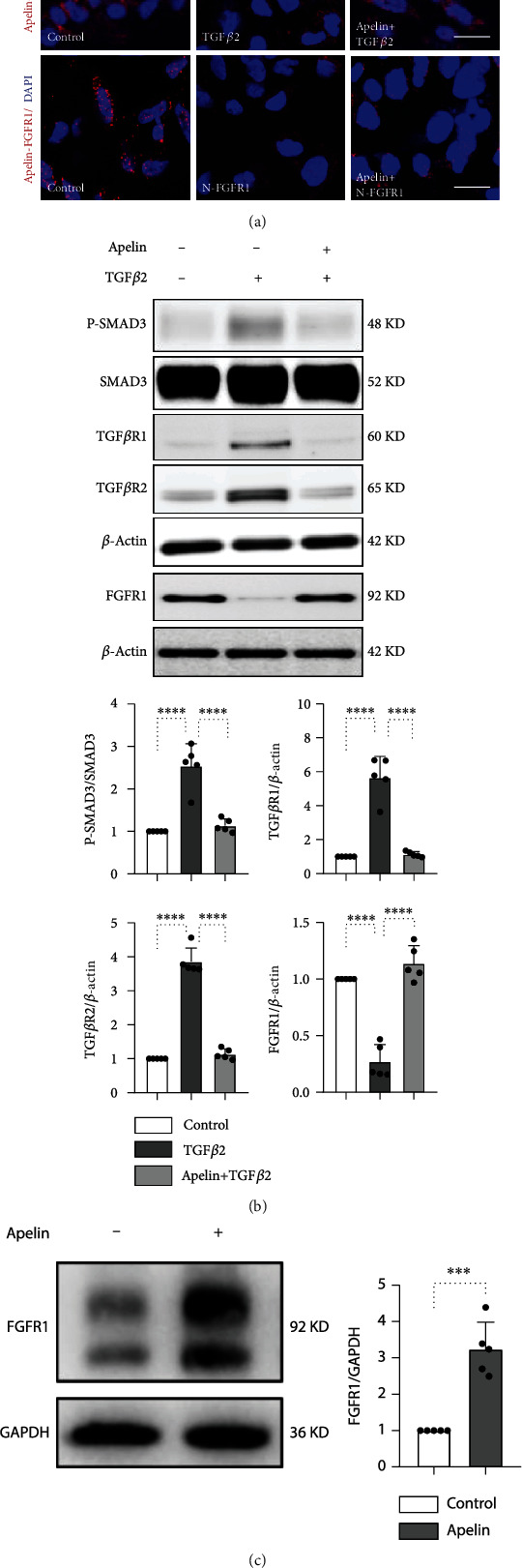
Proximity between apelin and FGFR1 suppresses the TGF*β*/Smad signaling pathway in HMVECs. (a) HMVECs were treated with TGF*β*2 (5 ng/mL) or N-FGFR1 (1.5 *μ*g/mL) for 48 h with or without preincubation with apelin (100 nM) for 2 h. The proximity between apelin and FGFR1 was then analyzed by the Duolink In Situ Assay. For each slide, images at a ×400 original magnification were obtained from six different areas. The scale bar is 60 *μ*m in each panel. (b) HMVECs were treated with TGF*β*2 (5 ng/mL) or with apelin (100 nM) for 48 h, and the p-Smad3, TGF*β*R1, TGF*β*R2, and FGFR1 levels were analyzed by western blot. Densitometric analysis of the p-Smad3/Smad3, TGF*β*R1/*β*-actin, TGF*β*R2/*β*-actin, and FGFR1/*β*-actin levels from each group (*n* = 5) was analyzed. (c) HMVECs were treated with or without apelin (100 nM) for 48 h, and the FGFR1 levels were analyzed by western blot. Densitometric analysis of the FGFR1/GADPH levels from each group (*n* = 5) was analyzed.

To examine the proximity between apelin and FGFR1 in cultured HMVECs, we performed Duolink *in situ* PLA. The results showed close proximity between apelin and FGFR1 in normal cultured HMVECs, indicating that endogenous apelin interacts with FGFR1 (Figure 1(a)). In the presence of a neutralizing FGFR1 antibody (N-FGFR1), the close proximity between apelin and FGFR1 was diminished in endothelial cells (Figure 1(a)). However, proximity was not restored by exogenous apelin incubation in the presence of N-FGFR1, suggesting that FGFR1 is a downstream target of apelin (Figure 1(a)). Western blot analysis showed that endogenous apelin restored TGF*β*2 and suppressed FGFR1 expression in HMVECs, indicating the inhibitory effect of TGF*β*2 on the apelin and FGFR1 complex (Figures 1(b) and 1(c)).

We then investigated whether exogenous apelin could suppress the TGF*β*/Smad signaling pathway in the endothelium. In the presence of TGF*β*2, the close proximity between apelin and FGFR1 was significantly diminished, and exogenous apelin incubation reversed this proximity, suggesting that TGF*β*2 inhibited the interaction between apelin and FGFR1 (Figure 1(a)). Apelin significantly inhibited TGF*β*2-induced Smad3 phosphorylation (p-smad3), TGF*β*R1, and TGF*β*R2 levels, which confirmed the inhibitory effect of apelin on TGF*β*/Smad signaling (Figure 1(b)). Additionally, exogenous apelin treatment significantly promoted the expression of FGFR1 in the endothelium (Figure 1(c)).

### 3.2. Apelin Inhibits TGF*β*/Smad Signaling and EndMT via Regulating FGFR1 Expression in Endothelial Cells

**Figure 2:**
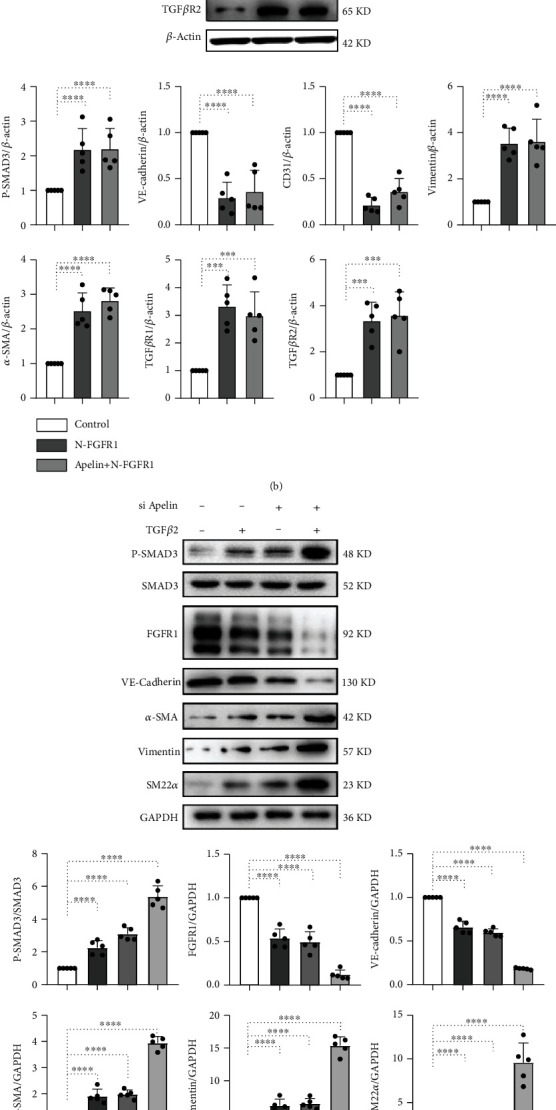
Apelin inhibits TGF*β*/Smad signaling and EndMT via regulating FGFR1 expression. (a) HMVECs were treated with TGF*β*2 (5 ng/mL) for 15 min or 48 h with or without preincubation with apelin for 2 h, and the VE-cadherin, CD31, *α*-SMA, SM22*α*, and FSP1 levels were analyzed by western blot. Densitometric analysis of the VE-cadherin/*β*-actin, CD31/*β*-actin, *α*-SMA/*β*-actin, SM22*α*/*β*-actin, and FSP1/*β*-actin levels from each group (*n* = 5) was analyzed. (b) HMVECs were incubated with N-FGFR1 (1.5 *μ*g/mL) in the presence or absence of apelin for 48 h, and the p-Smad3, VE-cadherin, CD31, vimentin, and *α*-SMA levels were analyzed by western blot. Densitometric analysis of the p-Smad3/Smad3, VE-cadherin/*β*-actin, CD31/*β*-actin, vimentin/*β*-actin, and *α*-SMA/*β*-actin levels from each group (*n* = 5) was analyzed. (c) HMVECs were transfected with or without apelin siRNA for 48 h in the presence or absence of TGF*β*2, and the p-smad3, FGFR1, VE-cadherin, *α*-SMA, vimentin, and SM22*α* levels were analyzed by western blot. Densitometric analysis of the p-Smad3/Smad3, FGFR1/GADPH, VE-cadherin/GADPH, *α*-SMA/GADPH, vimentin/GADPH, and SM22*α*/GADPH levels from each group (*n* = 5) was analyzed. (d) HMVECs were incubated with or without ML221 (apelin inhibitor, 10 *μ*M) for 48 h in the presence or absence of TGF*β*2, and the p-smad3, VE-cadherin, *α*-SMA, vimentin, and SM22*α* levels were analyzed by western blot. Densitometric analysis of the p-Smad3/Smad3, VE-cadherin/GADPH, *α*-SMA/GADPH, vimentin/GADPH, and SM22*α*/GADPH levels from each group (*n* = 5) was analyzed.

To further study the role of apelin in the regulation of TGF*β*-induced EndMT, western blotting was performed to detect protein levels of Smad3, p-Smad3, FGFR1, and EndMT markers. Exogenous apelin treatment significantly increased CD31 and VE-cadherin levels and decreased SM22*α*, FSP1, and *α*-SMA levels in cultured HMVECs. In addition, TGF*β*2-induced EndMT was reversed by exogenous apelin treatment, which verified that apelin suppressed TGF*β*-induced EndMT (Figure 2(a)).

Previous studies have confirmed that the inhibition of FGFR1 activates TGF*β*/Smad signaling and induces EndMT [6, 37]. We then investigated whether apelin inhibited TGF*β*/Smad signaling and EndMT by regulating FGFR1 expression. N-FGFR1 treatment significantly increased p-Smad3 expression and EndMT in endothelial cells, and apelin could not reverse N-FGFR1-induced Smad3 activation and EndMT, suggesting that the anti-EndMT and anti-TGF*β*/Smad effects of apelin were lost in FGFR1-deficient cells (Figure 2(b)).

TGF*β*2 has been shown to be a strong activator of Smad3 and downstream EndMT [37], and endothelial apelin knockdown showed a consistent effect. siRNA-mediated knockdown of apelin increased p-Smad3 expression and EndMT in endothelial cells, especially when TGF*β*2 and apelin siRNAs were applied together (Figure 2(c)). An apelin inhibitor (ML221) also showed a similar result, suggesting that inhibition of apelin downregulated FGFR1 and promoted TGF*β*-induced EndMT (Figure 2(d)).

### 3.3. Conditioned Medium from Apelin-Knockdown HUVECs Mediates the Induction of EMT in HK2 Cells

**Figure 3:**
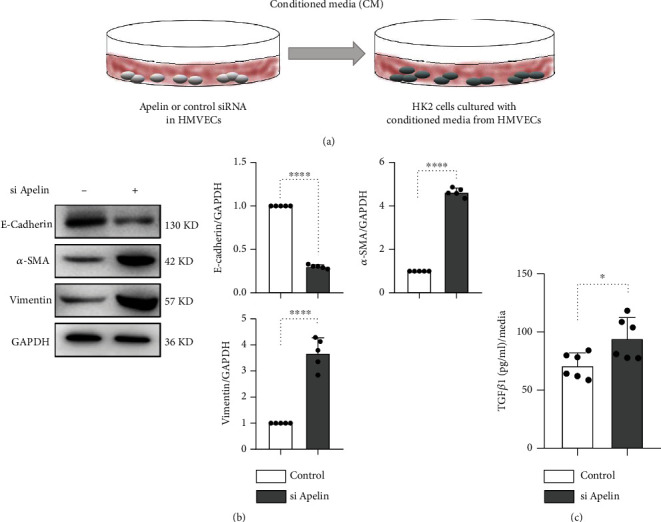
Apelin deficiency stimulates neighboring epithelial cells to transition into mesenchymal cells. (a) Design of the conditional medium experiment. HUVECs were transfected with scramble or apelin siRNA, and after 6 h, the medium was replaced with fresh medium and incubated for 48 h. After incubation of fresh medium for 48 h, the experimental media of HUVECs were harvested and transferred to cultured HK2 cells. (b) HK2 cells incubated with conditioned medium from apelin-knockdown HMVECs for 48 h, and the E-cadherin, vimentin, and *α*-SMA levels were analyzed by western blot. Densitometric analysis of the E-cadherin/GADPH, *α*-SMA/GADPH, and vimentin/GADPH levels from each group (*n* = 5) was analyzed. (c) ELISA analysis of TGF*β*1 levels from conditioned medium.

To address whether apelin deficiency in the endothelium could influence the extent of EMT in renal tubular cells, we established a conditioned medium experiment to test whether apelin knockdown could enhance EMT in HK2 cells, an immortalized human proximal tubule epithelial cell line (Figure 3(a)). The experimental medium from apelin siRNA-transfected HUVECs, but not that from scramble siRNA-transfected HUVECs, significantly decreased E-cadherin levels and increased vimentin and *α*-SMA levels in HK-2 cells, indicating that apelin knockdown in endothelial cells induced EMT in tubular epithelial cells (Figure 3(b)). We also measured TGF*β*1 levels in the medium of apelin or scramble siRNA-transfected HUVECs, and apelin knockdown remarkably increased TGF*β*1 levels compared to those in the control group (Figure 3(c)).

### 3.4. CEBPA Knockdown Promotes TGF*β*-Mediated EndMT

**Figure 4:**
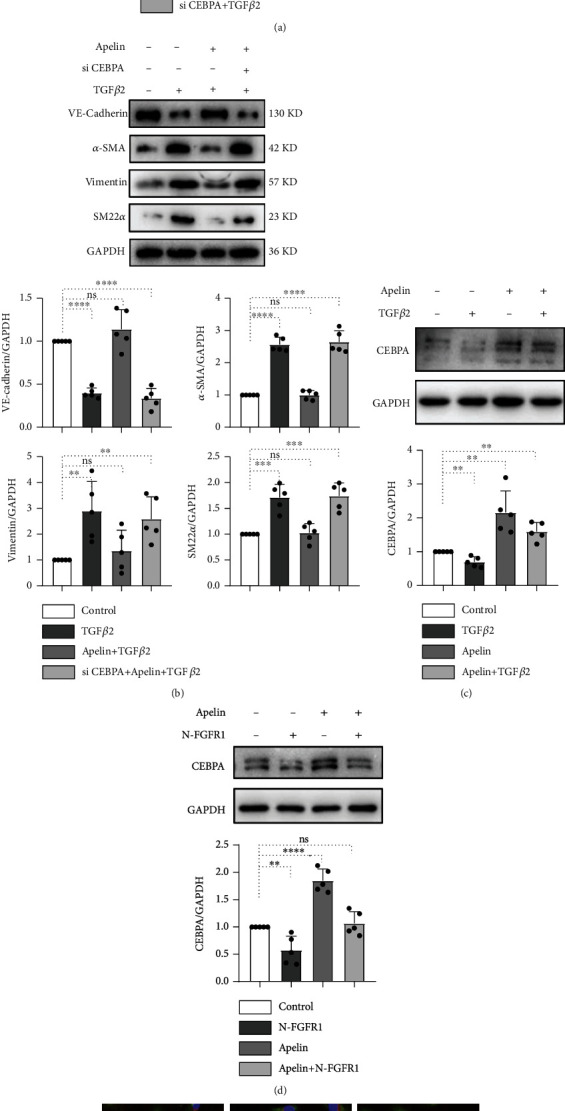
CEBPA knockdown promotes TGF*β*-mediated EndMT. (a) HMVECs were transfected with or without CEBPA siRNA for 48 h in the presence or absence of TGF*β*2, and the VE-cadherin, *α*-SMA, vimentin, and SM22*α* levels were analyzed by western blot. Densitometric analysis of the VE-cadherin/GADPH, *α*-SMA/GADPH, vimentin/GADPH, and SM22*α*/GADPH levels from each group (*n* = 5) was analyzed. (b) HMVECs were transfected with CEBPA siRNA for 48 h in the presence or absence of TGF*β*2 and apelin, and the VE-cadherin, *α*-SMA, vimentin, and SM22*α* levels were analyzed by western blot. Densitometric analysis of the VE-cadherin/GADPH, *α*-SMA/GADPH, vimentin/GADPH, and SM22*α*/GADPH levels from each group (*n* = 5) was analyzed. (c) HMVECs were treated with TGF*β*2 for 15 min or 48 h with or without preincubation with apelin for 2 h, and the CEBPA levels were analyzed by western blot. Densitometric analysis of the CEBPA/GADPH level from each group (*n* = 5) was analyzed. (d) HMVECs were treated with N-FGFR1 for 48 h or 15 min in the presence or absence of apelin, and the CEBPA levels was analyzed by western blot. Densitometric analysis of the CEBPA/GADPH level from each group (*n* = 5) was analyzed. Immunofluorescence analysis of CD31 (e) and *α*-SMA (f) coexpression in HUVECs following TGF*β*2 or/and apelin or/and CEBPA siRNA treatment. For each slide, images of six different fields of view at ×200 magnification were evaluated. The scale bar is 50 *μ*m in each panel.

It has been reported that CEBPA (C/EBP*α*) is a Smad3-repressed target during TGF*β*-induced EMT in human breast cancer [41, 42]. To further evaluate the effect of CEBPA on endothelial cells, western blotting and immunofluorescence were performed to detect the expression of EndMT markers. Knockdown of CEBPA significantly decreased VE-cadherin levels and increased the expression of *α*-SMA, vimentin, SM22*α*, and exogenous TGF*β*2, and/or apelin did not influence the expression levels of EndMT markers (Figures 4(a) and 4(b)). In addition, CEBPA expression was inhibited in the presence of TGF*β*2, whereas exogenous apelin treatment significantly increased CEBPA expression. When TGF*β*2 and apelin were applied together, the CEBPA expression level was slightly increased compared to that in the control group (Figure 4(c)). To verify the interaction between apelin, the FGFR1 complex, and CEBPA, HUVECs were treated with N-FGFR1 and apelin. CEBPA was significantly suppressed in the presence of N-FGFR1, and apelin restored the inhibitory effect of FGFR1 deficiency (Figure 4(d)). Immunofluorescence analysis also showed that the loss of CEBPA induced EndMT in the endothelium, and the expression of CD31, VE-cadherin, and *α*-SMA was not affected by exogenous TGF*β*2 and/or apelin treatment, indicating that CEBPA is a downstream target of apelin/TGF*β* signaling (Figures 4(e) and 4(f)).Taken together, these results strongly indicate that depletion of apelin and the FGFR1 complex could impair CEBPA expression, and the TGF*β*-induced repression of CEBPA expression contributed to the initiation of EndMT.

### 3.5. Apelin Inhibits Diabetes-Induced EndMT In Vivo

**Figure 5:**
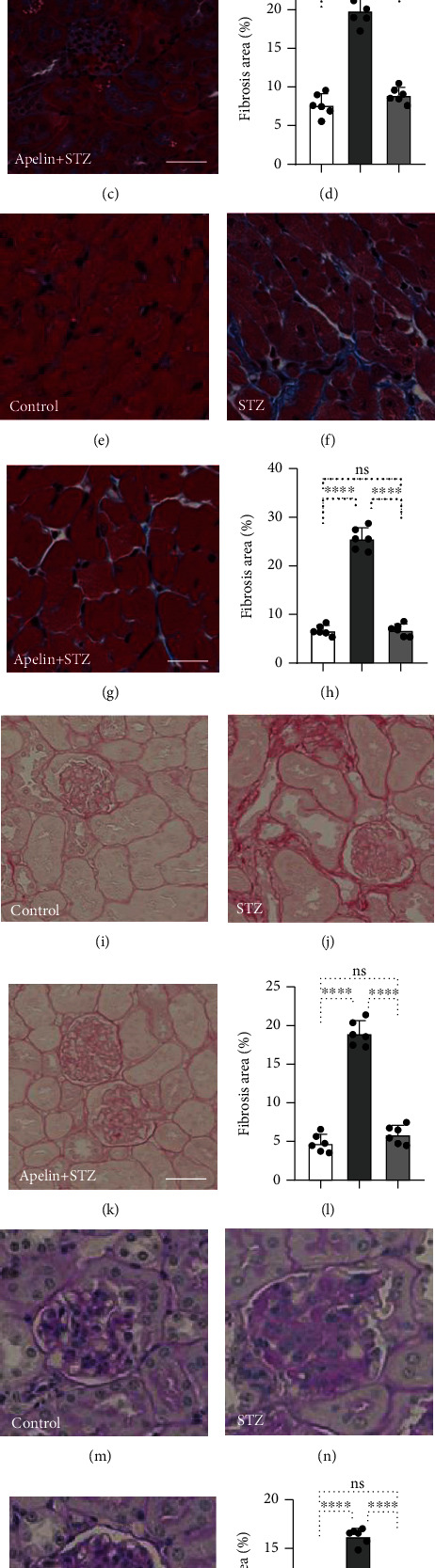
Apelin suppresses organ fibrosis and ameliorates diabetes-induced glomerular damage. Masson's trichrome staining (MTS) in the control (a), diabetic (b), and apelin-treated diabetic kidney (c). Scale bar: 40 *μ*m. Quantification of relative fibrosis areas was calculated using the ImageJ software (d). MTS in the control (e), diabetic (f), and apelin-treated diabetic heart (g). Scale bar: 40 *μ*m. Quantification of relative fibrosis areas was calculated using the ImageJ software (h). Sirius red staining in the control (i), diabetic (j), and apelin-treated diabetic kidney (k). Scale bar: 40 *μ*m. Quantification of relative fibrosis areas was calculated using the ImageJ software (l). Periodic acid-Schiff (PAS) staining in the control (m), diabetic (n), and apelin-treated diabetic kidney (o). Scale bar: 60 *μ*m. Quantification of the relative areas of glomeruli by the ImageJ software (p). For each section, images from six different fields of view at ×400 original magnification were obtained.

To identify the anti-EndMT and antifibrosis effect of apelin in vivo, we established the apelin-treated streptozotocin- (STZ-) induced diabetic mouse model. Masson's trichrome staining (MTS) results showed that the collagen fibers, stained blue, were significantly increased in diabetic kidney, suggesting massive organ fibrosis in diabetic mice (Figures 5(a) and 5(b)). However, the kidney and heart tissues of apelin-treated diabetic mice showed minor fibrotic alterations (Figures 5(c) and 5(d)). The similar phenomenon was observed in heart tissues that fibrotic alterations were ameliorated with the treatment of apelin in diabetic mice (Figures 5(e)–5(h)). In the kidneys, Sirius red staining demonstrated that the red-colored collagen was markedly increased, consistent with the MTS results (Figures 5(i)–5(l)). Periodic acid-Schiff (PAS) staining was used to evaluate the glomerular structure and damage in diabetic kidneys. The results showed that apelin inhibited ECM deposition, collagen accumulation, and glomerulosclerosis in the kidneys of apelin-treated diabetic mice (Figures 5(m)–5(p)).

**Figure 6:**
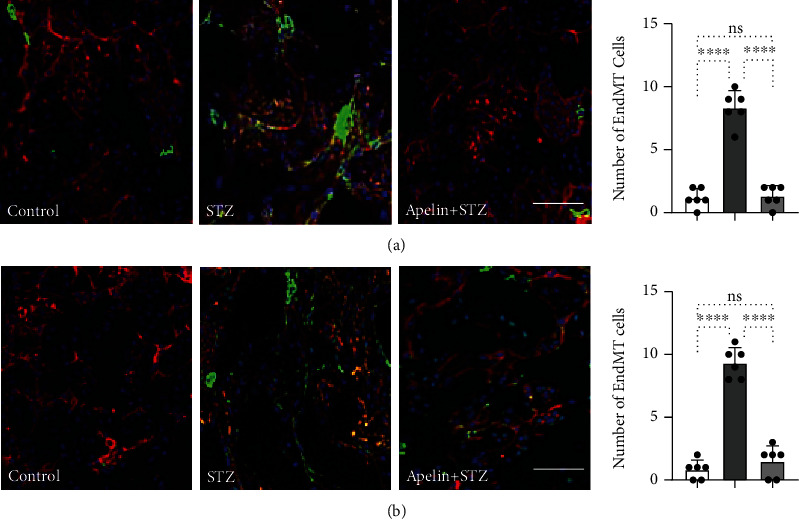
Apelin inhibits EndMT in diabetic kidneys. Immunofluorescence microscopy analysis of CD31/FSP1 (a) and CD31/*α*-SMA (b) in the kidney tissues from each group of mice. The scale bar is 40 *μ*m in each panel. The CD31 and FSP1 double-positive cells, the CD31 and *α*-SMA double-positive cells, and the CD31 and vimentin double-positive cells were recognized as the cells undergoing EndMT. For each section, images from six different fields of view at ×400 original magnification were obtained. And the cells undergoing the EndMT were counted and quantified by the ImageJ software.

In addition, we analyzed endothelial cells in the kidneys undergoing EndMT by co-immunolabeling of FSP1 and *α*-SMA with CD31. Compared with the control group, diabetic kidneys showed more endothelial cells undergoing EndMT, suggesting that apelin remarkably suppressed diabetes-induced endothelial damage (Figure 6). Moreover, the mRNA level of other fibrosis markers and other TGF beta signaling molecules was not remarkably influenced by the treatment of apelin, like fibronectin, collagen-1, collagen III, SMAD-2, and SMAD-4 (supplementary Figure 1(a, b)). Apelin suppressed the level of miR-29 and promoted the level of miR-let-7 in both diabetes kidneys and HK2 cells (supplementary Figure 1(c, d)).

## 4. Discussion

In this study, we described the anti-EndMT and antifibrotic effects of apelin via the inhibition of TGF*β*/Smad signaling. *In vitro*, we confirmed that apelin inhibited TGF*β*-induced EndMT by increasing the abundance of FGFR1 in the endothelium, and conditioned medium from apelin-knockdown HUVECs mediated EMT activation in HK2 cells. Activation of TGF*β*/Smad suppresses CEBPA expression, which mediates the initiation of EndMT in endothelial cells. *In vivo*, apelin ameliorated diabetes-induced renal fibrosis and glomerular damage, and the anti-EndMT effect of apelin was confirmed in endothelial cells of diabetic kidneys.

Fibroblast activation and parenchyma loss are the main characteristics of organ fibrosis [43, 44]. It has been reported that the apelin pathway is involved in the progression of pathological and physiological fibrosis, including kidney fibrosis, myocardial fibrosis, hepatic fibrosis, and pulmonary fibrosis [17, 43, 45]. Previous studies have reported that FGF/FGFR1 suppresses TGF*β*-induced EndMT, which results in the proliferation of fibroblasts and deposition of the extracellular matrix [37, 46]. Although there is evidence of a microRNA-dependent correlation between apelin and FGF2/FGFR1 pathways in pulmonary artery endothelial cells [36], the molecular interactions between apelin and FGFR1 in TGF*β* signaling are unclear. Our results showed that the close proximity between apelin and FGFR1 inhibited TGF*β*/Smad signaling. The increased abundance of apelin inhibited TGF*β*-induced EndMT by upregulating FGFR1 expression in the endothelium (Figure 1). FGFR1 deficiency reversed the anti-EndMT effect of apelin by targeting TGF*β*/Smad signaling (Figures 1 and 2). A reverse effect was confirmed by the knockdown of apelin at the mRNA and protein levels (Figure 3).

Apelin inhibits TGF*β*-dependent EMT in tubular epithelial cells to attenuate renal interstitial fibrosis [32]. Additionally, exogenous apelin has been shown to restore the EMT of podocytes, and decreased abundance of *β*5i enhanced the anti-EMT effect of apelin in podocytes in diabetic mice [33]. A recent study showed that the EndMT effect on peritubular capillaries could be induced by the EMT phenotype in proximal tubular cells through soluble factors [8]. Our results demonstrated that apelin deficiency in endothelial cells induced EMT in tubular epithelial cells, indicating that the EndMT phenotype of endothelial cells alters the phenotype of normal tubular epithelial cells (Figure 4).

Emerging evidence has shown that apelin exerts a protective effect against DN [23, 33, 47, 48]. Chen et al. and Day et al. reported that apelin acts as a suppressor of diabetes-induced inflammation, renal hypotrophy, and glomerular expansion in mice with type 1 diabetes [47, 48]. Liu et al. and Yin et al. confirmed the inhibitory effects of apelin on autophagy and EMT in podocytes of diabetic mice [23, 33]. Our study confirmed that apelin exhibited antifibrotic and anti-EndMT effects in endothelial cells in diabetic kidneys and hearts and remarkably ameliorated diabetes-induced glomerular damage (Figures 5 and 6). miR-29 and miR-let-7 were confirmed to contribute to pathological processes including kidney fibrosis [49]. Our results demonstrated that apelin suppressed the level of miR-29 and promoted the level of miR-let-7 (supplementary Figure 1).

CEBPA has been confirmed to be a Smad3-repressed target in TGF*β-*induced EMT during tumorgenesis. In the presence of TGF*β* treatment, nuclear Smad3 acts as a transcriptional repressor of CEBPA, and depletion of C/EBP*α* expression promotes the activation of EMT [41]. Similarly, we found that TGF*β*-induced repression of CEBPA expression contributed to the initiation of EndMT. Apelin and the FGFR1 complex impaired EndMT by targeting the TGF*β*/Smad/CEBPA axis (Figure 4).

## 5. Conclusion

In this study, we focused on the anti-EndMT and antifibrotic effects of apelin, which are generally thought to be important for kidney protection. First, the proximity of apelin and FGFR1 is critical for suppression of the TGF*β*/Smad signaling pathway in the endothelium. Second, apelin inhibits TGF*β*/Smad signaling and EndMT by regulating FGFR1 expression in endothelial cells. We found that the conditioned medium from apelin-knockdown HUVECs mediated the induction of EMT in HK2 cells. In addition, CEBPA knockdown promotes TGF*β*-mediated EndMT. In vivo, apelin inhibited diabetes-induced EndMT. These data provide clear evidence of the anti-EndMT and antifibrotic effects of apelin via the inhibition of TGF*β*/Smad signaling.

In conclusion, the results of this study revealed that the interaction of apelin and FGFR1 displayed renoprotective potential through suppression of the TGF*β*/Smad/CEBPA-mediated EndMT/EMT pathway.

## Data Availability

The data used to support the findings of this study are available from the corresponding author upon request.

## Supplementary Materials

Supplementary Materials Materials and methods of supplementary figure: RNA extraction and qPCR. Total RNA was isolated using the miRNeasy kit (Qiagen) and quantified using a NanoDrop spectrophotometer (NanoDrop Technologies, DE, USA), according to the manufacturer's instructions. Complementary DNA (cDNA) was generated using a miScript II RT kit (Qiagen) and the HiSpec buffer method. miR expression was quantified using the miScript SYBR Green PCR Kit (Qiagen) with 3 ng of complementary DNA. Total RNA (50 ng of total RNA was reverse-transcribed using a TaqMan miRNA Reverse Transcription Kit with primers for miR-29, let-7, and Hs_RNU6-2_1 (Applied Biosystems). This was followed by an RT-PCR using a standard TaqMan microRNA assay protocol with TaqMan probes for these miRNAs. All experiments were performed in triplicates, and Hs_RNU6-2_1 was used as the internal control. Supplementary Figure 1: apelin inhibits EndMT not via angiogenesis protein and inflammatory protein in diabetic kidneys. (a) The collagen I, collagen III, fibronectin, Smad2, and Smad4 levels were analyzed by qPCR in the kidney tissues from each group of mice. (b) The VEGFR1 and VEGFR2 levels were analyzed by qPCR in the kidney tissues from each group of mice. (c) The levels of miR29 and miR-let-7s were analyzed by qPCR in the kidney tissues from each group of mice. (d) HK2 cells incubated with high glucose medium with or without the incubated with apelin for 48 h, and the levels of miR29 and miR-let-7s were analyzed by qPCR.
