# Supplementary figures and images for: The Interaction of Apelin and FGFR1 Ameliorated the Kidney Fibrosis through Suppression of TGFβ-Induced Endothelial-to-Mesenchymal Transition
Source: Oxid Med Cell Longev. 2023 Feb 4;2023:5012474. doi: 10.1155/2023/5012474 (PMC9922196; doi:10.1155/2023/5012474)

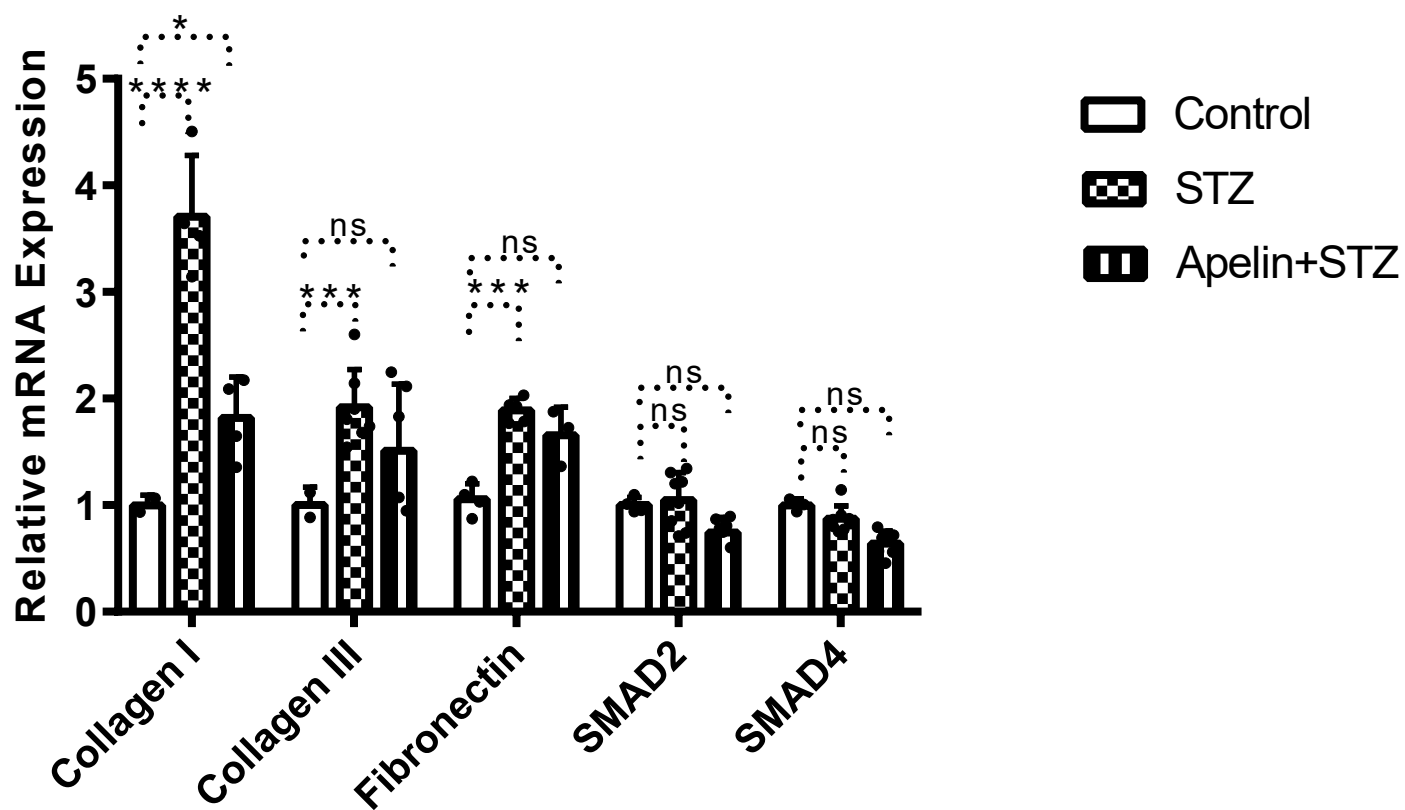

(a)

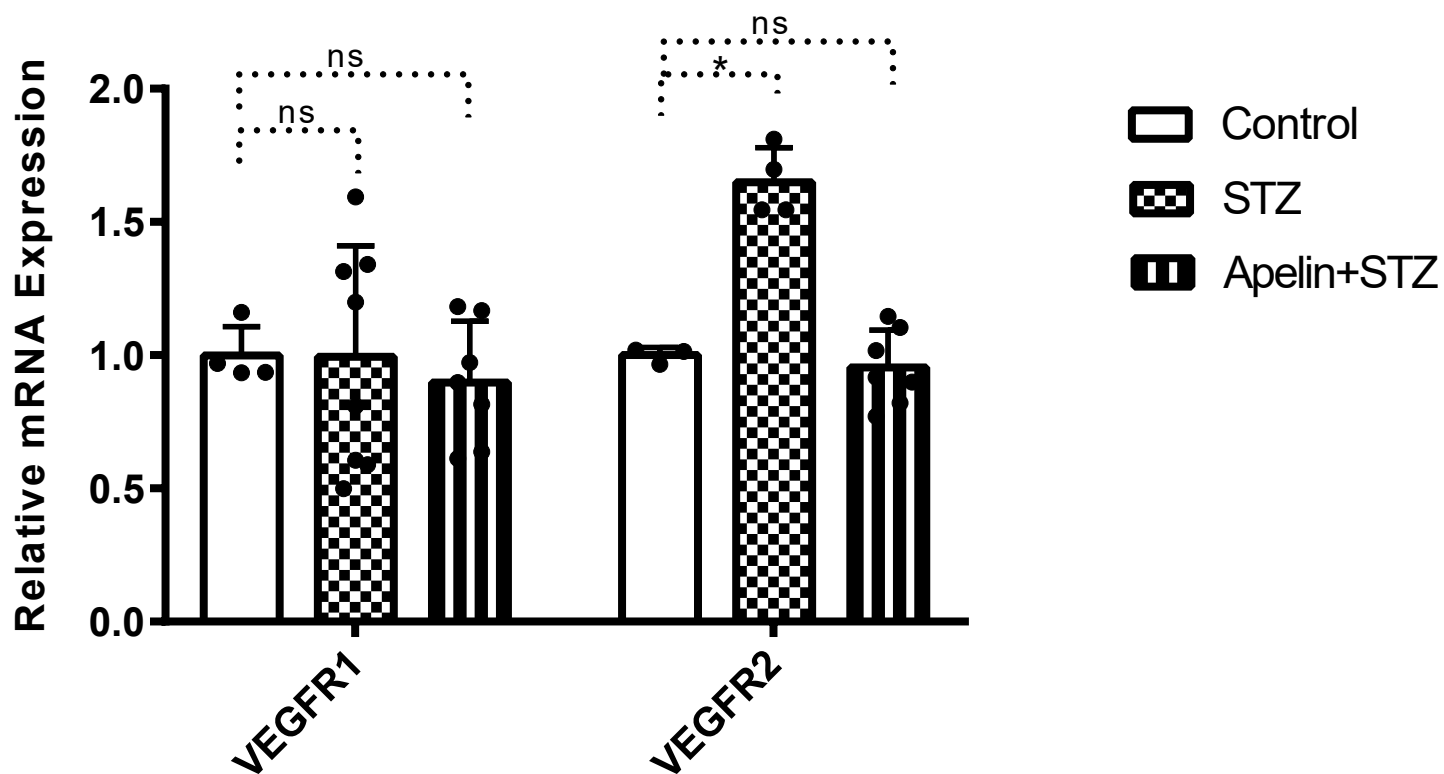

(b)

Supplement: Supplementary Materials — Materials and methods of supplementary figure: RNA extraction and qPCR. Total RNA was isolated using the miRNeasy kit (Qiagen) and quantified using a NanoDrop spectrophotometer (NanoDrop Technologies, DE, USA), according to the manufacturer's instructions. Complementary DNA (cDNA) was generated using a miScript II RT kit (Qiagen) and the HiSpec buffer method. miR expression was quantified using the miScript SYBR Green PCR Kit (Qiagen) with 3 ng of complementary DNA. Total RNA (50 ng of total RNA was reverse-transcribed using a TaqMan miRNA Reverse Transcription Kit with primers for miR-29, let-7, and Hs_RNU6-2_1 (Applied Biosystems). This was followed by an RT-PCR using a standard TaqMan microRNA assay protocol with TaqMan probes for these miRNAs. All experiments were performed in triplicates, and Hs_RNU6-2_1 was used as the internal control. Supplementary Figure 1: apelin inhibits EndMT not via angiogenesis protein and inflammatory protein in diabetic kidneys. (a) The collagen I, collagen III, fibronectin, Smad2, and Smad4 levels were analyzed by qPCR in the kidney tissues from each group of mice. (b) The VEGFR1 and VEGFR2 levels were analyzed by qPCR in the kidney tissues from each group of mice. (c) The levels of miR29 and miR-let-7s were analyzed by qPCR in the kidney tissues from each group of mice. (d) HK2 cells incubated with high glucose medium with or without the incubated with apelin for 48 h, and the levels of miR29 and miR-let-7s were analyzed by qPCR. [file 5012474.f1.zip › figureS1-1.pdf]

### kidney tissue

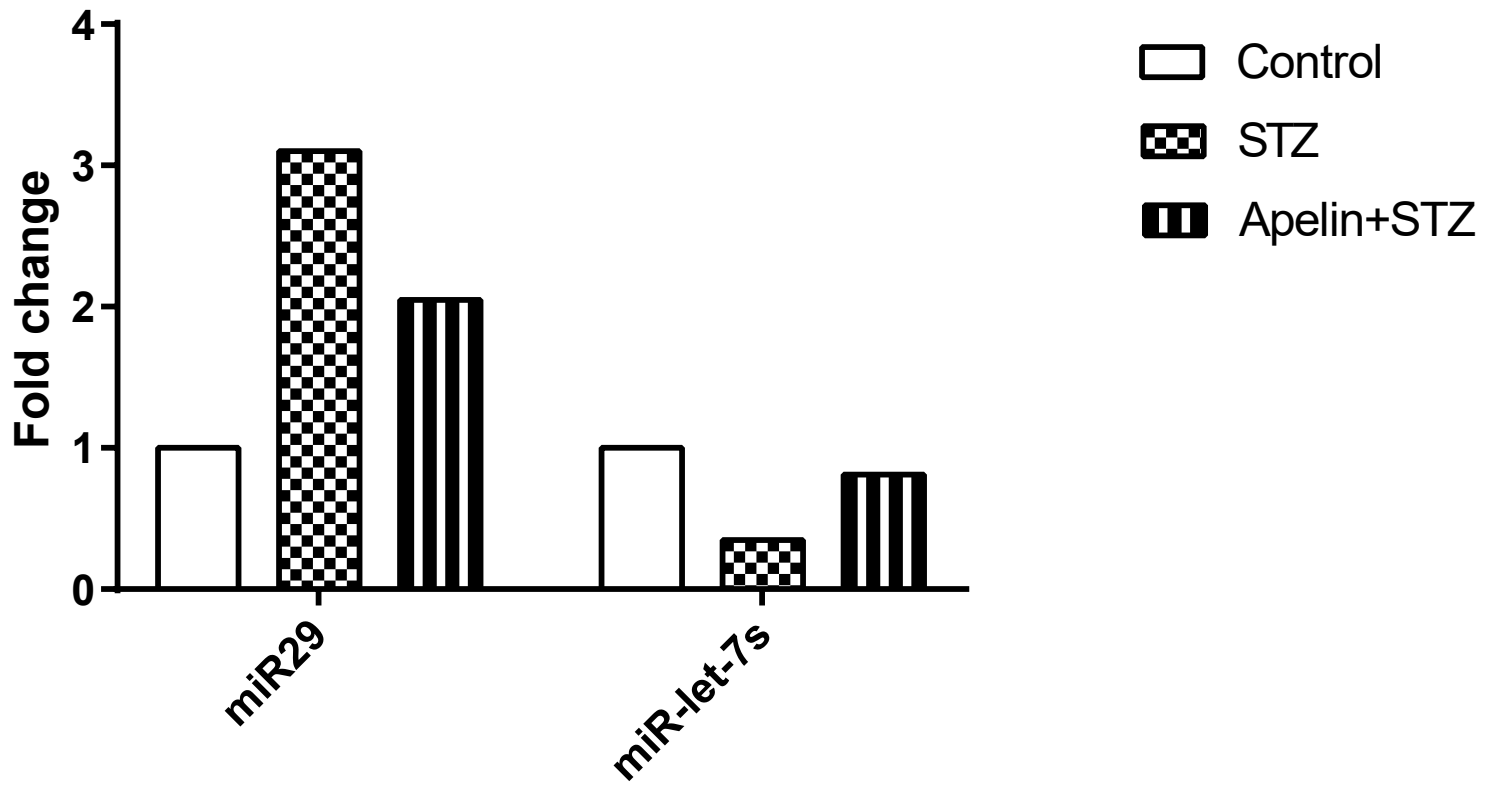

(c)

### HK2 cell

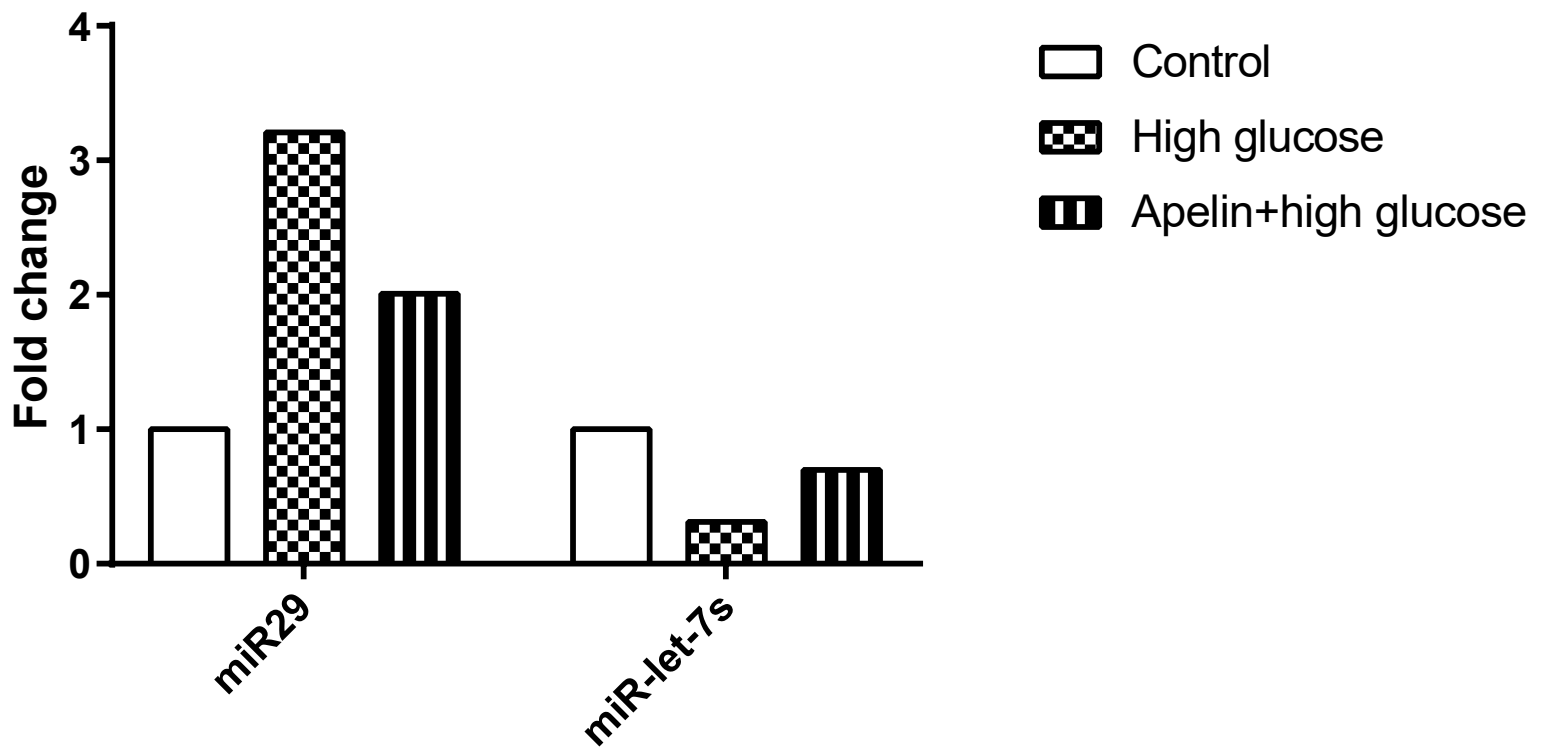

(d)

Supplement: Supplementary Materials — Materials and methods of supplementary figure: RNA extraction and qPCR. Total RNA was isolated using the miRNeasy kit (Qiagen) and quantified using a NanoDrop spectrophotometer (NanoDrop Technologies, DE, USA), according to the manufacturer's instructions. Complementary DNA (cDNA) was generated using a miScript II RT kit (Qiagen) and the HiSpec buffer method. miR expression was quantified using the miScript SYBR Green PCR Kit (Qiagen) with 3 ng of complementary DNA. Total RNA (50 ng of total RNA was reverse-transcribed using a TaqMan miRNA Reverse Transcription Kit with primers for miR-29, let-7, and Hs_RNU6-2_1 (Applied Biosystems). This was followed by an RT-PCR using a standard TaqMan microRNA assay protocol with TaqMan probes for these miRNAs. All experiments were performed in triplicates, and Hs_RNU6-2_1 was used as the internal control. Supplementary Figure 1: apelin inhibits EndMT not via angiogenesis protein and inflammatory protein in diabetic kidneys. (a) The collagen I, collagen III, fibronectin, Smad2, and Smad4 levels were analyzed by qPCR in the kidney tissues from each group of mice. (b) The VEGFR1 and VEGFR2 levels were analyzed by qPCR in the kidney tissues from each group of mice. (c) The levels of miR29 and miR-let-7s were analyzed by qPCR in the kidney tissues from each group of mice. (d) HK2 cells incubated with high glucose medium with or without the incubated with apelin for 48 h, and the levels of miR29 and miR-let-7s were analyzed by qPCR. [file 5012474.f1.zip › figureS1-2.pdf]
